# State-of-the-art cytometry in the search of novel biomarkers in digestive cancers

**DOI:** 10.3389/fonc.2024.1407580

**Published:** 2024-05-29

**Authors:** Carolina G. de Castro, Alejandro G. del Hierro, Juan H-Vázquez, Sara Cuesta-Sancho, David Bernardo

**Affiliations:** ^1^ Mucosal Immunology Lab, Institute of Biomedicine and Molecular Genetics (IBGM), University of Valladolid and Consejo Superior de Investigaciones Científicas (CSIC), Valladolid, Spain; ^2^ Centro de Investigaciones Biomedicas en Red de Enfermedades Infecciosas (CIBERINFEC), Madrid, Spain

**Keywords:** spectral cytometry, mass cytometry, colorectal cancer, hepatocellular carcinoma, computational cytometry, biomarkers, gastric cancer

## Abstract

Despite that colorectal and liver cancer are among the most prevalent tumours in the world, the identification of non-invasive biomarkers to aid on their diagnose and subsequent prognosis is a current unmet need that would diminish both their incidence and mortality rates. In this context, conventional flow cytometry has been widely used in the screening of biomarkers with clinical utility in other malignant processes like leukaemia or lymphoma. Therefore, in this review, we will focus on how advanced cytometry panels covering over 40 parameters can be applied on the study of the immune system from patients with colorectal and hepatocellular carcinoma and how that can be used on the search of novel biomarkers to aid or diagnose, prognosis, and even predict clinical response to different treatments. In addition, these multiparametric and unbiased approaches can also provide novel insights into the specific immunopathogenic mechanisms governing these malignant diseases, hence potentially unravelling novel targets to perform immunotherapy or identify novel mechanisms, rendering the development of novel treatments. As a consequence, computational cytometry approaches are an emerging methodology for the early detection and predicting therapies for gastrointestinal cancers.

## Graphical Abstract

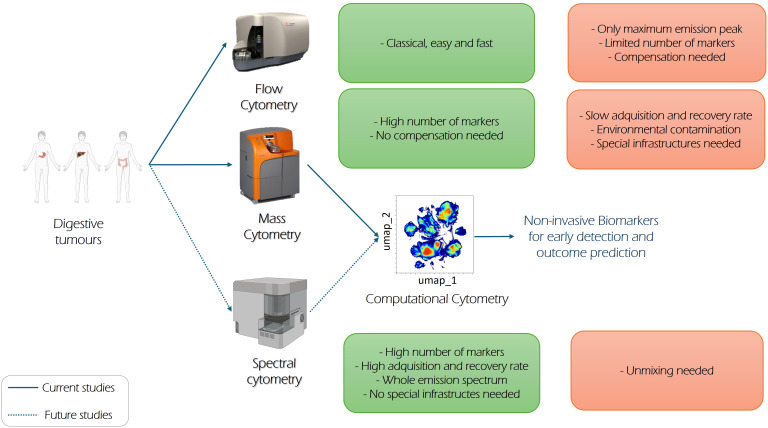

## 1 Digestive tumours

Currently, and according to the International Agency for Research on Cancer (1), the most prevalent cancers in the world are lung, breast, colorectal, prostate, stomach, and liver cancers, respectively. Although lung cancer is currently the one with the highest mortality, tumours within the gastrointestinal tract (colorectal, liver, and stomach) are the most prevalent ones when considering overall incidence and mortality. Among them, colorectal cancer (CRC) is actually the second global cause (1) of cancer-related mortality. Most of the CRC cases are adenocarcinoma, and 60%–65% of them are sporadic, 25% have a CRC family case without any hereditary syndrome, and 5% of them are caused by a cancer hereditary syndrome such as non-polyposis colorectal cancer (or Lynch syndrome) and familial adenomatous polyposis (FAP). Lynch syndrome is caused by mutations in genes encoding mismatch DNA repairing proteins, while FAP is due to mutations in the tumour suppressor gene *APC*. Moreover, colorectal cancer can be subdivided based on microsatellite stability (MSS) and microsatellite instability (MSI) due to this deficiency of mismatch repairing proteins. MSI tumours have better prognosis, as they can be easily treated with immunotherapy than MSS tumours (2–4). While there are more several factors such as aging or location (right side CRC has worse survival than the left side) (5) underlying its development, the most relevant risk contributors include modifiable elements such as obesity, sedentary lifestyle, low fruit and vegetable intake, red meat consumption, and smoking and alcoholic habits. Its frequency is higher in economically developed countries, although it is also increasing in westernised developing societies (6–8). Indeed, in 2022, colorectal cancer was the most diagnosed tumour and the second leading cause of cancer death in Europe (9). Its 5-year survival rate ranges from 10% to 90% depending on the stage at diagnose with a better prognosis if it is diagnosed at earlier stages. Screening methods include faecal occult blood test or double contrast barium enema, which are subsequently followed (if positive) by a colonoscopy (10).

Gastric cancer is the fifth most common tumour (1) in the world, and, as CRC, most of them are adenocarcinoma (11). It is usually classified as cardia gastric (upper stomach) and non-cardia gastric cancer (mid and distal stomach) having both different risk factors and epidemiological patterns (12). Of the gastric cancer cases, 90% are due to environmental factors while the remaining 10% have a familiar component. Hereditary diffuse gastric cancer, which has a worse survival than sporadic gastric tumours, is commonly caused by germinal mutations in *CDH1* gene, which codes E-cadherin protein. Hereditary Peutz–Jeghers syndrome predisposes to early-onset gastric cancer. Lynch syndrome also increases the lifetime risk of developing it (13–17). Whereas non-cardia gastric cancer is related to smoking and deficient diet, cardia class correlates with obesity and gastroesophageal reflux disease (18). Despite overall predisposing factors including dietary factors (like high consumption of processed meats, smoked foods, and high salt diets) (19), *Helicobacter pylori* infection is responsible for almost 90% of distal gastric cancers. Indeed, the International Agency for Research on Cancer labels this bacterium as a human carcinogen due to its correlation with gastric cancer, as it provokes gastritis, which can derive on stomach atrophy, metaplasia, and dysplasia. Its 5-year survival rate for early stages is 75.4% (20), so screening analysis, such as esophagogastroduodenoscopy, are usually implemented.

Last, but not the least, liver cancer remains the sixth most common tumour (1) worldwide. As opposed to both colorectal and gastric cancer, this one is a remarkable heterogeneous disease that has been redefined over time. However, hepatocellular carcinoma is its most frequent histological manifestation appearing in approximately 90% of the cases (21). Risk factors include alcohol abuse, diabetes, exposure to aflatoxin, and genetic predisposition. Nevertheless, almost 80% (22) of hepatocellular carcinoma are associated with hepatitis B/C virus infection. In addition, high-risk groups include patients with non-alcoholic fatty liver and cirrhosis. The Barcelona Clinic for Liver Cancer (BCLC) classifies hepatocellular carcinoma status and determine treatments options based on it (from 0 to D). Another common indicator is the MELD model (Model for End-stage Liver Disease), which examines the bilirubin, creatinine, and International Normalised Ratio (INR) from each patient to predict the 3-month mortality with a statistical confidence of 80%, in order to prioritise those patients who need an earlier transplant (23). Although early stages of hepatocellular carcinoma are treatable, only 20% of diagnosed patients survive more than 1 year. Liquid biopsies such as cell-free DNA or α-fetoprotein are being studied as potential early detection biomarkers, but further research is required (22, 24, 25).

Given therefore the aggressive nature of digestive tumours, there is no doubt that early diagnosis is still the best weapon to fight them. In this regard, the identification of novel biomarkers can provide novel tools not only to aid on their diagnosis and predict their subsequent prognosis (26–30) but also unveil novel targets to perform immune therapy. However, although this has been partially achieved in the gastric one given the role that *H. pylori* infection elicits on its trigger, such goal remains elusive for both CRC and liver cancer.

Regarding hepatocellular carcinoma (HCC), alpha-fetoprotein (AFP) is widely used combined with abdominal ultrasound in order to determine HCC illness. However, it is also true that targeted therapies are essential for the treatment of hepatocellular carcinoma (HCC), yet tackling this type of cancer is challenging owing to its molecular heterogeneity. Some predictive biomarkers based on gene polymorphisms (like VEGFD), serum protein levels, or clinical predictors such as hypertension can be used to identify responders to sorafenib or levantinib, both of which serve as first-line systemic therapies (31). However, it is obvious that more studies are needed to obtain new biomarkers with utility in the clinical practice.

Related to CRC, clinicians focus on DNA signatures to design treatment options. As previously stated, high MSI is the phenotype of 15% colorectal tumours. These tumours typically do not metastasise and show poor response to chemotherapy. Despite this, they have a better clinical course than MSS tumours because of the higher production of neoantigens, making them more susceptible to treatment with immunotherapy (32). Other therapeutic strategies focus on the MAPK pathway status. RAS, which controls cells proliferation, is often mutated in CRC (33), and monoclonal antibodies (mAbs) anti-EFGR as cetuximab are typically used to stop the abnormal signalling. Other mAbs like encorafenib targets KRAF, which is usually mutated in CRC, and it is used when anti-EFGR are not effective, as it is downstream in the MAPK pathway (32). Focusing on liquid biopsies, several serum proteins such as carcinoembryonic antigen (CEA) are found in CRC. However, they do not correlate significantly with prognosis or survival rates. New approaches also investigate circulating tumour cells and circulating tumour DNA in the blood. In contrast with CEA and other serum proteins, these markers correlate with treatment response and survival rates, making them desirable biomarkers to study in depth before starting a therapeutic option (32, 34, 35).

Building from the current needs, this manuscript will therefore focus on the utility that flow cytometry elicits on the search of novel biomarkers with aid on the clinical practise of digestive tumours.

## 2 Conventional and mass cytometry

Conventional flow cytometry (CFC) is a fluorescence-based technique, which allows the immunotyping of immune cells at single-cell resolution, which has been used in the clinical setting for over 50 years (36). In addition, it is currently used for B- and T-cell leukaemia and lymphoma immunophenotyping, helping to identify their optimal treatment. However, given that CFC is based on the use of fluorochrome-labelled specific antibodies, the maximum number of fluorochromes—and therefore markers—which can be analysed by classical CFC, is restricted to the number of available channels in the equipment. Therefore, conventional cytometers in the clinical setting do not usually provide more than 8 or 10 channels, hence limiting the number of parameters that can be analysed. Although that has been recently overridden by the development of novel equipment, which display up to 20 channels and, therefore, can identify up to 20 markers at the same time (37), the development of CFC panels using more than 15 markers simultaneously requires advanced skills to compensate the fluorescence among the different markers. In addition, fluorochromes with a similar emission wavelength or close emission peaks cannot be combined in the same panel, hence limiting the capacity to perform complex panels.

In order to overcome these limitations, mass cytometry or CyTOF (cytometry time-of-flight) allows the analysis of over 40 markers at the single-cell level. That can be achieved because, as opposed to CFC, antibodies are tagged with heavy metals (like stable lanthanide isotopes) instead of fluorochromes, so cells are characterised based on atomic weight (38). Therefore, mass cytometry has proven to be highly valuable in the deep characterisation of immune cells (39) given its ability to identify over 40 parameters within a single cell (40, 41), rendering it a “pseudo-omic” technique (38), which has allowed the identification of novel biomarkers (42–46).

## 3 Computational cytometry

The capacity, however, of developing complex panels with over 40 parameters in a single-cell comes with a cost. Although flow cytometry data analyses are traditionally performed following a hierarchical gating strategy of serial selections in two-dimensional plots, this approach is time consuming, subjective, and can be also influenced by the operator’s experience and biases. In addition, the amount of obtained data with complex panels is exponentially increased. Therefore, when addressing complex panels, it becomes obvious that novel unbiased approaches are required in order to address the whole variability (47, 48). Thus, computational approaches based on unsupervised algorithms have emerged to address such complexity (49).

To that end, preliminary data cleaning (either with an unsupervised cleaning algorithm or with manual removal) is essential to remove outlier events due to abnormal flow behaviours resulting from clogs and other common technical problems. Once that has been performed, a subsequent unbiased exploratory approach can be performed on the dataset in an efficient and reproducible way. Dimensionality reduction algorithms, which are commonly used to perform an unsupervised and exploratory cytometry analysis, organise the data based on the protein expression levels, projecting all the variables into two or three dimensions. T-distributed Stochastic Neighbour Embedding (t-SNE) focuses on the non-linear differences between cells in protein marker expression to create a two-dimension map retaining the local data structure. However, the global distances are not reliable, and the number of cells that it can support is limited (50). Hence, it is common to perform a randomised method of event downsizing to a given dataset called “Downsampling” or “Subsampling”. Although this entails the possibility of losing under-represented populations along the way, it is possible to perform a subsequent unsupervised analysis by directly addressing the previously identified populations of interest, allowing the use of a larger number of events from these populations, which would confer greater robustness to the results. In addition, it is possible to implement the hierarchical analysis as a way of validating the results obtained, thus avoiding the loss of information that would involve doing the process in reverse (50, 51). Hierarchical Stochastic Neighbour Embedding (H-SNE) overcomes t-SNE because it allows to achieve dimension reduction algorithm without subsampling, becoming a solid and powerful single-cell analysis method (52). Given that t-SNE quadratically scales its computation time in function of the number of cells being analysed, another algorithm option is the Uniform Manifold Approximation and Projection (UMAP), which has a faster computation time than t-SNE, preserves better the global data structure, and attaches additional data to an existing plot, reasons why it has a powerful clinical monitoring impact (for instance, comparing a treatment efficacy) (50). However, such approaches have certain issues, such as the absence of a standardised and agreed-upon pipeline protocol (due to his novelty) or the inability to eliminate subjectivity in the analysis steps and the technical limitations. Although computational cytometry approaches become more widespread, they are a highly computationally demanding method (50, 51).

In spite of that, results obtained by computational cytometry approaches are data-driven and operator-independent, hence increasing their reproducibility, comparability, and accuracy, which, altogether, allow the identification of novel cell subsets related to a specific condition that otherwise may have gone unnoticed (50, 51) so they can be further analysed (and isolated) following directed hierarchical gating approaches.

## 4 Mass cytometry and colorectal cancer

Mass cytometry and computational cytometry have been used in several studies involving mouse models to better understand patient’s cancer and improve their therapeutic options. For instance, although PD-1/PD-L1 is a promising immunotherapy based on the inhibition of these immune checkpoints, not all patients derive benefit from this treatment; hence, it may need to be enhanced by other immune checkpoint blockade (ICB). Beyrend et al. (53) generated a CRC mouse model treated with PBS or PD-L1 therapy, and their tumour-infiltrating lymphocytes (TILs) were analysed by computational and mass cytometry. They found that the PD-L1 group showed a distinct T-cell subset expressing LAG-3 (inhibitory molecule) and ICOS (activating molecule). These findings suggest that enhancing antitumour immunity could be achieved by targeting LAG-3 or ICOS. Moreover, Krieg et al. (54) found that the complement component 3a receptor (C3aR) was downregulated in patients with CRC independently of their MSS or MSI status. Despite the role of the C3a–C3aR interaction in maintaining homeostasis and creating a tumour-free microenvironment, their CRC model lacking C3aR demonstrated that their protumourogenic faecal microbiota induced a significant immune infiltrate, which could be targeted with ICB. In addition, macrophages in CRC, known as tumour-associated macrophages (TAMs), can develop a proinflammatory (M1) or anti-inflammatory (M2) phenotype, leading this last one to T-cell exhaustion and poor prognosis (55). MS4A4A is a transmembrane protein related to these events, and it serves as a biomarker for the M2 phenotype (56); however, the role in CRC of TAMs and MS4A4A remains unclear. Using murine models, Li et al. (57) showed that MS4A4A induces polarisation towards the M2 phenotype, leading to T-cell exhaustion and a poor prognosis. Blocking MS4A4A with anti-MS4A4A detained CRC progression and enhanced the efficacy of anti-PD-1 therapy.

On this wise, Tang et al. (58) found that colon cancer mice injected with IFN-γ and anti-PD-1 had reduced tumour growth and less amount of M2 macrophages; however, they also observed an upregulation of LAG-3 in response to anti-PD-1 therapy.

Oncolytic virus therapy is a kind of nanomaterial used to treat cancer. Zhang et al. (59) designed a protein nanocage with the structure of hepatitis B virus and CpG motifs, which are immunostimulatory. Using a CRC murine model, they found by mass cytometry that after being exposed to the nanocages, the tumour microenvironment was modified having a higher expression of CD8 (cytotoxic) T cells and decreasing the T-cell exhaustion. The treatment led to enhanced antitumour immunity.

Specifically focused in the human setting, the application of novel and complex multiparametric panels in the CRC setting by CyTOF has proved that there is a huge immune diversity between different patients and within each individual, not only between the blood and the intestinal immune infiltrate but also between the affected and non-affected tissue (60). In addition, certain circulating immune cells such as monocytes and NK cells subsets are altered during perioperative period (61), confirming that CRC requires a personalised approach.

Mass cytometry has, for instance, proved that the pregnane X receptor mediates the mechanisms of oxaliplatin therapy resistance in tumour cells by eliminating the drug from the cells (62). In addition, and following the first cycle of oxaliplatin chemotherapy, patients reduce their total NK cell numbers due to a specific decrease in the CD56^dim^CD16^−^ subset. In a similar manner, the CD16^+^ NK fraction was also reduced due the presence of several proinflammatory cytokines (IL-2, IL-15, and IL-18), which ultimately leads to lose their cytotoxic activity (63). In addition, Ting Zhang et al. (64) have shown that although EpCAM^+^CD4^+^ T-cell subsets can infiltrate the tumour in a CCR5- and CCR6-dependent manner, they nevertheless displayed an exhausted and immunosuppressive phenotype, as they were both not only PD-1^+^ but also PD-L1^+^. Not surprisingly, and given its immune-suppressive effect, such cell subset has become the target for immune checkpoint blockade therapies. In addition, Yang Luo et al. (65) found that high levels of circulating T-cell co-expressing CD103^+^CD39^+^ predict clinical response to such immune checkpoint inhibitors.

In line with the above, Tsunenori et al. (66) proved that IL-6 produced by stromal and tumour cells correlated with lower T_reg_ (CD4^+^FOXP3^+^) cells in the tumour microenvironment. In addition, IL-6 serum levels positively correlated with enhanced levels of myeloid-derived suppressor cells (MDSCs) and effector regulatory T-cells (eT_reg_), two types of immunosuppressive cell subsets, while high levels of IL-6 in the tumour microenvironment correlates with immune evasion and a debilitated antitumour activity. In a similar manner, eT_reg_ (defined as BLIMP-1^+^FOXP3^+^) were increased in the tumour infiltrate. These cells can be further divided into FOXP3^lo^ and FOXP3^hi^. In addition, higher levels of the first subset in the tumour infiltrate correlate with good outcomes while the later correlate with bad prognosis. Therefore, and given that eT_reg_ cells have been associated with both good and bad prognosis in CRC, it seems obvious that further investigation is required in order to specifically identify the specific subset, which can be used as a therapeutic target (67).

Cytokeratin 20 (CK20) is a common biomarker used to determine CRC tumour stage and histological grade. In addition, its expression on tumour cells is related to poor prognosis, whereas its absence indicates a less-invasive cancer (68). Despite that, terminally differentiated epithelial cells have a lower proportion of CK20 expression in CRC tissues referred to controls, while its expression also differs, within CRC patients, based on the tumour microsatellite stability. This reveals the necessity of fully understanding the colorectal cancer status of the patients in each of its different stages (69).

On the other hand, it is obvious that the gut microbiota can also modulate the outcome of immune responses in CRC (70). Indeed, mucosa-associated invariant T cells (MAIT) cells recognise bacterial riboflavin antigens following presentation by the non-classic histocompatibility complex MR1. Building from that, Shamin Li et al. (71) found that tumour-infiltrating MAIT cells are CD39^+^ and display an increased expression of exhaustion markers PD-1^+^ and CTL4^+^ coupled with lower cytokine production. Together, these suggest that chronic activation of MAIT cells induces the expression of CD39 together with senescence markers leading to T-cell exhaustion. In addition, *Fusobacterium nucleatum* induces CD39 expression on MAIT in a TCR-dependent manner, hence providing a mechanism by which such bacteria are bad prognosis factors in CRC.

One of the most advantageous locations for identifying biomarkers are liquid biopsies like peripheral blood where we have PBMCs. Kong et al. (72) studied the differences between immune subsets during CRC development. Through mass cytometry and computational analysis, they revealed that central memory CD4^+^ T cells, switched B cells, CD16^−^ NK cells, monocytes, and basophils were increased in adenocarcinoma compared with adenoma and healthy controls, while double-negative T cells were decreased in adenoma and adenocarcinoma. Moreover, effector CD4^+^ T cells and naive B cells were higher in CRC patients with lymph node metastasis, while basophils and unswitched B cells were lower.

Mass cytometry can also monitor the effects of a clinical trial in the patient’s immune system. Monjazeb et al. (73) performed mass cytometry to evaluate the immune impact on the patients of a phase 2 trial of combined anti-CTLA-4 and anti-PD-1 therapy. They found that patient’s PBMCs had a lower amount of CD4^+^ and CD8^+^ T cells compared with patients treated with two different radiation regimens (with generally no differences between them). This included activation markers such as CXCR3 or ICOS.

Yang et al. (74) decided to use mass cytometry among other exploratory methods to study primary liver cancers and liver metastases from other cancers including CRC. They found that hepatic metastatic CRC had higher levels of CD4^+^ T_reg_, LAG-3^+^CD4^+^ T-cells, CD27^+^PD-1^+^CD8^+^ T-cells, and lower levels of CD57^+^PD-1^+^CD8^+^ T cells than non-hepatic metastatic CRC. Circulating levels of this immune subsets have therefore the potential to be considered as novel biomarkers to predict patients with a higher probability of subsequently developing metastases.

## 5 Mass cytometry and liver cancer

Mass cytometry has also been implemented in the context of liver cancer and its main variant, hepatocellular carcinoma (HCC). The full comprehension of HCC illness becomes complicated due to its heterogeneity. In addition, although there is a wide range of different treatments based on tumour stage, they however do not always translate into clinical remission (75); it appears mandatory to explore new targets and therapeutic options (21). Moreover, mass cytometry approaches can be also applied as diagnostic tools. Hence, a complex panel applied on blood samples can identify several immune disturbances, which can be used to allow an early detection of different solid tumours including HCC (56).

Animal models of HCC are often used to study this condition through mass cytometry. In the search of new proposing target markers for therapies, research in mice revealed that GDF15 protein induces enhancement of T_reg_ through CD48 (76), and this leads to immunosuppression in HCC; blockade of this protein could potentially be translated to human therapies. When studying effectiveness of treatments, it was found that SUMOylation levels are higher in HCC liver than in normal liver tissues. Subsequently, it was proved that inhibition of this process enhances antitumour activity and could be a therapeutic option (77). In another study using mice, it was found that “M2-like” tumour-associated macrophages are more prevalent in a specific type of mouse model resistant to anti-anti-PD-1 therapy (78). However, the conclusion highlights the wide diversity in the immune tumour microenvironments among preclinical models, in addition to the intrinsic heterogeneity of HCC in humans. It seems imperative to expand studies in mouse models to obtain translational results for humans. Therefore, in this review, we will focus on human studies.

Focused on the human setting, potential new therapeutic strategies could benefit from mass cytometry studies regarding HCC cells mechanisms. For example, it has been revealed that tumour cells and their intrinsic activation of β-catenin pathway results in the recruitment of MDSC and the formation of immune “desert” phenotype, which could be a potential treatment strategy option (79). However, immune system description regarding HCC involves not only novel immunotherapy targets but also prognosis and evolution biomarkers and cell subsets. Undeniably, the role of NK cells remain fundamental for immunity and especially in cancer (80). Indeed, CD56^+^ and CD56^dim^ NK cells may be useful as immunotherapies targets for HCC patients, as they have impairment phenotypes in hepatocarcinoma affected from different aetiologies, especially in non-alcoholic fatty liver disease (NAFLD) patients (21, 81).

In HCC, it is also possible to explore the patient’s immunity depending on the stage or type of development that they have achieved. Although patients with HCC who successfully had a transplant are scarce, they display a unique immune fingerprint, since mass cytometry approaches have shown that such differential immune profile can be used to stratify and predict patients with risk of recurrence, which can be very helpful when designing patient treatment (82).

Also building from mass cytometry results, it has been proven that the CD161^+^/CD161^−^ ratio within peripheral CD8^+^PD-1^+^ T cells predicts subsequent disease outcome with better prognosis with higher ratios (83). Specifically focused on the tissue (affected, non-affected, and border area), although PD-1 expression does not predict the outcome of the patients, it is actually the balance between tissue resident memory and PD-1^+^ exhausted T cells that is relevant for such outcome, since a high tissue resident memory/exhausted T-cell ratio in tumour microenvironment determines positive patient prognosis (84, 85). Better prognosis conditions are expected from HCC patients who have a non-terminally exhausted phenotype in tumour-resident memory T cells, which are specific to HBV response (86), and also for those patients with enriched CD4^+^CD8^+^ T cells, especially if they are expressing PD-1 and located in leading-edge regions (87).

When focusing on ongoing therapies, radiofrequency ablation is the primary first-line treatment option for HCC patients who are not eligible for surgery. Aiming to study its effects on tumour immune response, a mass cytometry study revealed that decreased levels of CD8^+^ effector and memory T cells, among others, correlated with a worst immune response against tumour cells (88). As for current therapy options, immune checkpoint inhibitors (including PD-1/PD-L1) have reached long-term response rates of 14%–20% in HCC patients. However, there is no information about patients who would benefit from this treatment due to its aetiology. In addition, CyTOF analyses revealed that neither viral aetiology nor the current viral status in HCC patients were variables to consider when deciding to prescribe PD-1 inhibitor therapy (89). On the other hand, the use of threonine tyrosine kinase inhibitor changes the proportion of circulating immune subsets before following treatment, with bigger changes on patients with a better outcome (90). Another scenario in which immune activation occurs, leading to a favourable response in HCC patients, is Yttrium90-Radioembolisation treatment (91). There are other types of uncommon treatments under study, such as the use of compound 2,5-dimethylcelecoxib (92), which has proven an increased infiltrate of NK cells within HCC tumours, resulting in favourable prognosis and suggesting its potential as a therapy target.

In summary, HCC pathology, heterogeneity, and treatment response have been broadly studied by CyTOF. Some immune cell subsets like CD56 NK cells or the CD161^+^/CD161^−^ ratio within CD8^+^PD-1^+^ T cells may indeed be useful to stratify patients based on their aetiology and their subsequent outcome, while other subsets can predict clinical response to immune checkpoint inhibitors.

## 6 Future perspectives and remarks

Mass cytometry has undoubtedly shown a great potential in the study of human gastrointestinal tumours. Nevertheless, it is also true that although this approach overcomes most of the CFC approaches, it also has some specific handicaps (41, 93).

The main one is the low acquisition rate, as it only allows to acquire approximately 500 cells per second instead of more than 10,000 in CFC. In addition, it is an expensive technique, since the costs of not only the equipment but also the special reagents and labelling compounds are significantly higher than those in flow cytometry. In a similar manner, cell vaporisation is an irreversible process and restricts the potential for subsequent *ad hoc* sorting. Likewise, due to lower cell recovery, there is a decrease in sensitivity for detecting low abundance proteins. Last, but not the least, as with a classical sorter, the equipment requires operation by a highly trained person, hence limiting its widespread accessibility.

An alternative to overcome these limitations is spectral cytometry, which combines the principal features of CyTOF while abrogating its handicaps. Therefore, as opposed to the CFC, spectral cytometry provides a measure of the entire fluorescence emission spectrum. That way, classical issues associated with CFC compensation and autofluorescence issues are reduced through a concrete unmixing algorithm (94). In addition, as it uses fluorochromes, reagents are usually cheaper, since it is not the same ones used in CFC. Moreover, its acquisition speed is high (up to 30,000 events per second) (95). Overall, these are even more attractive features, especially considering that comparable results can be expected from both procedures (96).

However, both spectral and mass cytometry data must offer high-quality, reliable, and robust information to be considered valid. Hence, a careful panel design and validation, titration, and optimisation of antibodies and reference controls are required. Having said that, and given the properties of mass cytometry, it is also true that the implementation of novel panels is usually faster in that approach, since no unmixing controls are required to optimise a panel. Either way, sample staining on both approaches should always include normalisation control and correct equipment handling (97). On the contrary, both mass and spectral cytometry are capable of performing complex panels of up to 40 parameters (47). As a consequence, all the known immune cell subsets can be identified in a given sample by using these techniques. Hence, the ability to obtain such a wide range of information from each analysis provides a major reason to implement these approaches, spectral or mass cytometry, in various types of studies, especially in those aimed at discovering new biomarkers needed for some illnesses. In this regard, **Table 1** summarises the different aspects to take into consideration before considering mass or spectral cytometry approaches. Therefore, it seems obvious that spectral cytometry overtakes the approaches performed by mass cytometry, as it is faster, cheaper, and requires a smaller sample size. Indeed, there are several issues that need to be addressed in the near future for both CRC and HCC, and they may be overcome when using different types of approaches, for example spectral cytometry studies, which, to the best of our knowledge, have not been performed in this context.

**Table 1:** Conventional mass and spectral cytometry features.

	Conventional Flow Cytometry	Mass Cytometry	Spectral Cytometry
**Parameters capability**	Up to 30	>60	Up to 50
**Cell labelling**	Fluorochrome	Heavy metal	Fluorochrome
**Cell throughout**	>10,000 events/second	500 events/second	30,000 events/second
**Detection**	Fluorescence (maximum peak emission)	Mass spectrometry	Fluorescence (whole spectrum)

In addition, these techniques provide a high-resolution single-cell analysis, so the more parameters can be detected on cancer cells, the more specific will be the exploration of the tumour heterogeneity. The classification of the function and phenotype of the immune cells leads to an accurate examination of the altered cellular process, early diagnosis and detection, and prediction of therapy response. The integration of cytometry, proteomics, and genomics technologies will bring a promising precision medicine in order to improve patients’ outcomes and life expectancy (98).

## 7 Conclusions

The only current approach for CRC diagnosis and monitoring is the use of regular colonoscopies, which, however, are not only invasive and uncomfortable for the patients but also expensive and time consuming for the health systems. On the other hand, regarding HCC, most of its current knowledge has been obtained from murine models, which, although essential to deepen our understanding on such disease, they may not always translate into the human setting (99, 100). Therefore, given the large amount of information that computational cytometry approaches provide, these approaches will allow the identification of novel (and better) biomarkers to aid on CRC or HCC diagnosis, monitoring, or even predicting disease outcome. In addition, given that both mass and spectral cytometry usually focus on the study of the immune system, they may not only identify biomarkers but also provide novel insights into the specific pathogenic mechanisms potentially unravelling novel targets to perform immunotherapy or identify novel mechanisms, hence rendering to the development of novel treatments.

## Author contributions

CG: Methodology, Writing – original draft. AG: Methodology, Writing – original draft. JH: Methodology, Writing – original draft. SC: Supervision, Writing – review & editing. DB: Supervision, Writing – review & editing.
